# Association of Neighborhood-Level Household Income With 21-Gene Recurrence Score and Survival Among Patients With Estrogen Receptor–Positive Breast Cancer

**DOI:** 10.1001/jamanetworkopen.2023.0179

**Published:** 2023-02-21

**Authors:** Sung Jun Ma, Jasmin Gill, Olivia Waldman, Keerti Yendamuri, Cynthia Dunne-Jaffe, Udit Chatterjee, Fatemeh Fekrmandi, Rohil Shekher, Austin Iovoli, Song Yao, Oluwadamilola T. Oladeru, Anurag K. Singh

**Affiliations:** 1 Department of Radiation Medicine, Roswell Park Comprehensive Cancer Center, Buffalo, New York; 2 University at Buffalo, The State University of New York, Buffalo; 3 Jacobs School of Medicine and Biomedical Sciences, University at Buffalo, The State University of New York, Buffalo; 4 Department of Cancer Prevention and Control, Roswell Park Comprehensive Cancer Center, Buffalo, New York; 5 Department of Radiation Oncology, University of Florida, Gainesville

## Abstract

**Importance:**

While low income has been associated with a higher incidence of triple-negative breast cancer, its association with 21-gene recurrence score (RS) among patients with estrogen receptor (ER)-positive breast cancer remains unclear.

**Objective:**

To evaluate the association of household income with RS and overall survival (OS) among patients with ER-positive breast cancer.

**Design, Setting, and Participants:**

This cohort study used data from the National Cancer Database. Eligible participants included women diagnosed between 2010 and 2018 with ER-positive, pT1-3N0-1aM0 breast cancer who received surgery followed by adjuvant endocrine therapy with or without chemotherapy. Data analysis was performed from July 2022 to September 2022.

**Exposures:**

Low vs high neighborhood-level household income levels defined as below vs above the median household income of $50 353 based on each patient’s zip code.

**Main Outcomes and Measures:**

RS (a score ranged from 0 to 100 based on gene expression signatures indicating the risk of distant metastasis, with RS of 25 or below indicating non–high risk and RS above 25 indicating high risk) and OS.

**Results:**

Among 119 478 women (median [IQR] age, 60 [52-67] years; 4737 [4.0%] Asian and Pacific Islander, 9226 [7.7%] Black, 7245 [6.1%] Hispanic, 98 270 [82.2%] non-Hispanic White), 82 198 (68.8%) and 37 280 (31.2%) patients had high and low income, respectively. Logistic multivariable analysis (MVA) showed that, compared with high income, low income was associated with higher RS (adjusted odds ratio [aOR], 1.11; 95% CI, 1.06-1.16). Cox MVA showed that low income was also associated with worse OS (adjusted hazards ratio [aHR], 1.18; 95% CI, 1.11-1.25). Interaction term analysis showed a statistically significant interaction between income levels and RS (interaction *P* < .001). On subgroup analysis, significant findings were noted among those with RS below 26 (aHR, 1.21; 95% CI, 1.13-1.29), while there was no significant OS difference between income levels among others with RS of 26 or higher (aHR, 1.08; 95% CI, 0.96-1.22).

**Conclusions and Relevance:**

Our study suggested that low household income was independently associated with higher 21-gene recurrence scores and significantly worse survival outcomes among those with scores below 26, but not 26 or higher. Further studies are warranted to investigate the association between socioeconomic determinants of health and intrinsic tumor biology among patients with breast cancer.

## Key Points

**Question:**

Do household income levels factor into 21-gene recurrence score (RS) and mortality among patients with estrogen receptor (ER)-positive breast cancer?

**Findings:**

In this cohort study involving 119 478 women, low income was associated with higher RS and worse survival. On subgroup analysis, similar findings were observed among those with RS below 26, while there was no survival difference between income levels among others with scores 26 or higher.

**Meaning:**

These results suggest that low household income is associated with more aggressive tumor biology and significantly worse mortality among those with RS below 26.

## Teaser

This cohort study of US women with estrogen receptor (ER)-positive breast cancer examines the association of income levels with 21-gene recurrence scores and survival outcomes.

## Introduction

Socioeconomic status (SES) is measured using income, education, and occupational status and is a strong predictor of health outcomes.^1^ Low SES is a risk factor for unfavorable breast cancer outcomes regardless of racial and ethnic background.^2,3^ Decreased access to preventative care and cancer screening, with consequent effects on comorbidities of patients at their time of diagnosis presenting stage, have been implicated in the disparities associated with low SES.^3^ Differences in cancer treatment account for only a small fraction low SES disparities.^3^

Despite extensive research in the roles of socioeconomic inequities and breast cancer disparities, there is a paucity of studies evaluating the association of socioeconomic status with intrinsic tumor biology. For example, 21-gene recurrence score (RS), a score based on gene expression signatures indicating the risk of distant metastasis, has been incorporated into routine clinical care in the US.^4,5^ Although low SES and income levels are associated with a higher incidence of triple-negative breast cancer,^6,7,8^ the association between low income and RS among patients with estrogen receptor (ER)-positive breast cancer, the most common subtype, remains unclear. We performed an observational cohort study to evaluate the association of income levels with RS and survival among patients with nonmetastatic ER-positive breast cancer.

## Methods

This study was reviewed and approved by the institutional review board at the Roswell Park Comprehensive Cancer Center with an exemption for informed consent requirements because data were deidentified and publicly available. It followed the Strengthening the Reporting of Observational Studies in Epidemiology (STROBE) reporting guideline.

The National Cancer Database (NCDB) is a nationwide, clinical oncology database sponsored jointly by the American Cancer Society and the American College of Surgeons Commission on Cancer (CoC).^9^ It collected over 34 million cancer cases from more than 1500 CoC-accredited facilities,^9^ and it represented 80% of newly diagnosed breast cancer in the US.^10^ It also has been used for studies evaluating RS as shown in our previous studies.^11,12^ The database was queried for female patients diagnosed between 2006 and 2018 with ER-positive, pT1-3N0-1aM0 breast cancer who received surgery followed by adjuvant endocrine therapy. Patients were excluded if they had unknown RS or income levels. Follow-up was conducted until 2021. Other variables included for analysis were facility type, race and ethnicity, age, medical insurance, income and education level, Charlson-Deyo Comorbidity Score, year of diagnosis, histology, tumor grade, T and N staging, RS, lymphovascular space invasion, surgery, surgical margin, radiation therapy, and chemotherapy. Race and ethnicity were included as variables because they are considered factors in income levels and socioeconomic status. All missing values for each variable were grouped together and coded as unknown. Education and income levels were determined based on the 2016 American Community Survey data spanning years 2012 through 2016. It measured the percentage of adults who did not graduate from high school and the median household income adjusted for 2016 inflation, respectively, in each patient’s zip code and equally proportioned education and income ranges among all US zip codes. High vs low neighborhood-level income and education were determined by the median values of $50 353 and 10.9%, respectively, with the latter being the percentage of adults living in the zip code area who did not graduate from high school. Baseline characteristics were compared between those with known vs unknown RS and income levels. Other clinically relevant variables, including systemic therapy agents and duration, Karnofsky performance status, and breast cancer-specific survival, were not captured in the NCDB.

Our primary end points were RS and overall survival (OS). RS was defined as a score ranged from 0 to 100 indicating the risk of distant metastasis based on the gene expression signatures using pretreatment tumor specimens,^13^ with RS of 25 or below indicating non–high risk and RS above 25 indicating high risk for both node-negative^5^ and node-positive^4^ breast cancer.

OS was defined as the time interval between diagnosis and the last follow-up or death from any cause. Baseline characteristics were compared between high-income vs low-income levels using Fisher exact test and Mann-Whitney *U* test as appropriate. Logistic multivariable analysis (MVA) was constructed based on baseline patient and tumor characteristics as listed previously to evaluate variables associated with RS above 25. Cox MVA was used to evaluate OS. Cumulative incidence plots for overall mortality were generated. Survival data among patients diagnosed in 2018 were unavailable in the NCDB, and these patients were excluded for OS analysis. Cox MVA models included all variables listed previously.

Interaction term analysis was performed to evaluate any heterogeneous association between income levels and RS. If the interaction term was statistically significant, subgroup analyses were performed to compare the magnitude of income differences associated with OS stratified by RS. In order to reduce selection bias and further investigate the subgroup analysis results, propensity score matching between income levels was performed. Matching was based on all variables listed previously using nearest neighbor method in a 1:1 ratio with no replacements and a caliper distance of 0.25 of the standard deviation of the logit of the propensity score.^14^ Propensity scores were estimated using logistic regression models. To ensure adequate matching, standardized means differences for all matched variables were evaluated and calculated to be less than 0.1, suggesting negligible differences between income levels.^15^ Survival outcomes were reported after propensity score matching. Sensitivity analysis was also performed among non-Hispanic White women with high education level and private medical insurance in order to evaluate the association of income levels and OS.

All *P* values were 2-sided, and *P* < .05 was considered statistically significant. All analyses were performed using R version 4.0.3 (R Project for Statistical Computing).

## Results

A total of 119 478 women (median [interquartile range (IQR)] age, 60 [52-67] years; 4737 [4.0%] Asian and Pacific Islander, 9226 [7.7%] Black, 7245 [6.1%] Hispanic, 98 270 [82.2%] non-Hispanic White) met our criteria and were included for analysis (Table 1). Of these, 82 198 (68.8%) and 37 280 (31.2%) patients had high and low household income levels, respectively. Median (IQR) follow up was 66.2 (47.9-90.3) months. A total of 36 084 women did not meet our criteria for analysis. Most baseline characteristics were comparable between those with known vs unknown RS or income levels (eTable 1 in Supplement 1). However, most patients with unknown RS or income levels also had missing values for education levels.

**Table 1.** Baseline Characteristics Stratified by Income Levels

Characteristics	Income, No. (%)		*P* value
	High	Low	
Age, y			
<50	16 996 (20.7)	6259 (16.8)	<.001
≥50	65 202 (79.3)	31 021 (83.2)	
Facility			
Nonacademic	52 353 (63.7)	26 061 (69.9)	<.001
Academic	27 611 (33.6)	10 271 (27.6)	
Not available	2234 (2.7)	948 (2.5)	
Race			
Asian and Pacific Islander	3968 (4.8)	769 (2.1)	<.001
Black	3767 (4.6)	5459 (14.6)	
Hispanic	4525 (5.5)	2720 (7.3)	
Non-Hispanic White	69 938 (85.1)	28 332 (76.0)	
Insurance			
None	717 (0.9)	643 (1.7)	<.001
Private	52 399 (63.7)	18 882 (50.6)	
Government	28 249 (34.4)	17 388 (46.6)	
Not available	833 (1.0)	367 (1.0)	
Education^a^			
Above median	66 698 (81.1)	9497 (25.5)	<.001
Below median	15 500 (18.9)	27 783 (74.5)	
CDS			
0	71 138 (86.5)	30 193 (81.0)	<.001
1	9070 (11.0)	5540 (14.9)	
≥2	1990 (2.4)	1547 (4.1)	
Year			
2006-2013	32 908 (40.0)	15 160 (40.7)	.047
2014-2018	48 792 (59.4)	21 914 (58.8)	
Histology			
Ductal or lobular carcinoma	70 504 (85.8)	32 265 (86.5)	<.001
Other	11 694 (14.2)	5015 (13.5)	
PR			
Positive	74 507 (90.6)	33 661 (90.3)	.06
Negative	7691 (9.4)	3619 (9.7)	
T staging			
1	61 780 (75.2)	27 334 (73.3)	<.001
2	19 267 (23.4)	9402 (25.2)	
3	1151 (1.4)	544 (1.5)	
N staging			
0	69 896 (85.0)	31 571 (84.7)	.12
1a	12 302 (15.0)	5709 (15.3)	
Grade			
1	22 911 (27.9)	10 373 (27.8)	<.001
2	44 093 (53.6)	19 615 (52.6)	
3	12 176 (14.8)	5936 (15.9)	
Other	44 (0.1)	26 (0.1)	
Not available	2974 (3.6)	1330 (3.6)	
RS			
0-15	40 443 (49.2)	18 217 (48.9)	<.001
16-25	30 242 (36.8)	13 144 (35.3)	
>25	11 513 (14.0)	5919 (15.9)	
LVSI			
No	63 119 (76.8)	28 205 (75.7)	<.001
Yes	10 413 (12.7)	4231 (11.3)	
Not available	8666 (10.5)	4844 (13.0)	
Chemotherapy			
No	64 907 (79.0)	29 354 (78.7)	.38
Yes	17 291 (21.0)	7926 (21.3)	
Radiation			
No	25 148 (30.6)	12 030 (32.3)	<.001
Yes	56 132 (68.3)	24 838 (66.6)	
Not available	904 (1.1)	403 (1.1)	
Surgery			
Lumpectomy	55 886 (68.0)	24 900 (66.8)	<.001
Mastectomy	26 293 (32.0)	12 368 (33.2)	
Other	19 (<0.1)	12 (<0.1)	
Margin			
Negative	79 426 (96.6)	35 988 (96.5)	.37
Positive	2487 (3.0)	1144 (3.1)	
Not available	285 (0.3)	148 (0.4)	
Vital status			
Alive	78 156 (95.1)	34 505 (92.6)	<.001
Dead	4042 (4.9)	2775 (7.4)	

Abbreviations: CDS, Charlson-Deyo comorbidity score; LVSI, lymphovascular space invasion; PR, progesterone receptor; RS, 21-gene recurrence score.

^a^ Median education level was 10.9% of adults living in a zip code who did not graduate from high school.

Logistic MVA showed that, compared with high household income, low household income was associated with higher RS (adjusted odds ratio [aOR] 1.11, 95% CI, 1.06-1.16; *P* < .001) (Table 2). A total of 4042 (4.9%) and 2775 (7.4%) women with high-income and low-income levels died during the follow-up, respectively (Table 1). Cox MVA showed that low income was also associated with worse OS (adjusted hazards ratio [aHR], 1.18; 95% CI, 1.11-1.25; *P* < .001) (Table 3). Interaction term analysis showed a statistically significant interaction between income levels and RS (interaction *P* < .001).

**Table 2.** Multivariable Logistic Regression Analysis Results for High 21-Gene Recurrence Score

Characteristic	aOR (95% CI)	*P* value
Income		
Above median	1 [Reference]	[Reference]
Below median	1.11 (1.06-1.16)	<.001
Race		
Non-Hispanic White	1 [Reference]	[Reference]
Asian and Pacific Islander	1.04 (0.94-1.14)	.45
Black	1.19 (1.12-1.27)	<.001
Hispanic White	0.92 (0.85-0.99)	.04
Age, y		
<50	1 [Reference]	[Reference]
≥50	1.04 (0.99-1.10)	.15
Facility		
Nonacademic	1 [Reference]	[Reference]
Academic	0.96 (0.93-1.00)	.08
Insurance		
None	1 [Reference]	[Reference]
Private	1.08 (0.91-1.28)	.38
Government	1.03 (0.87-1.22)	.77
Education^a^		
Above median	1 [Reference]	[Reference]
Below median	1.04 (0.99-1.08)	.12
CDS		
0	1 [Reference]	[Reference]
1	1.02 (0.96-1.07)	.56
≥2	1.09 (0.98-1.21)	.11
Year		
For every 1 y increase	0.98 (0.97-0.99)	<.001
Histology		
Ductal or lobular carcinoma	1 [Reference]	[Reference]
Other	0.74 (0.70-0.78)	<.001
PR		
Positive	1 [Reference]	[Reference]
Negative	6.55 (6.25-6.87)	<.001
T staging		
1	1 [Reference]	[Reference]
2	1.31 (1.26-1.37)	<.001
3	0.8 (0.67-0.94)	.007
N staging		
0	1 [Reference]	[Reference]
1a	0.8 (0.75-0.84)	<.001
Grade		
1	1 [Reference]	[Reference]
2	2.76 (2.60-2.94)	<.001
3	16.83 (15.79-17.94)	<.001
Other	18.91 (11.32-31.43)	<.001
LVSI		
No	1 [Reference]	[Reference]
Yes	1.23 (1.17-1.30)	<.001

Abbreviations: aOR, adjusted odds ratio; CDS, Charlson-Deyo comorbidity score; LVSI, lymphovascular space invasion; PR, progesterone receptor.

^a^ Median education level was 10.9% of adults living in a zip code who did not graduate from high school.

**Table 3.** Multivariable Cox Regression Analysis Results for Overall Survival

Characteristics	aHR (95% CI)	*P* value
Income		
Above median	1 [Reference]	[Reference]
Below median	1.18 (1.11-1.25)	<.001
Race		
Non-Hispanic White	1 [Reference]	[Reference]
Hispanic White	0.8 (0.72-0.90)	<.001
Black	1.08 (1.00-1.18)	.05
Asian and Pacific Islander	0.66 (0.56-0.79)	<.001
Age, y		
<50	1 [Reference]	[Reference]
≥50	1.96 (1.77-2.17)	<.001
Facility		
Nonacademic	1 [Reference]	[Reference]
Academic	0.79 (0.75-0.84)	<.001
Insurance		
None	1 [Reference]	[Reference]
Private	0.64 (0.51-0.81)	<.001
Government	1.49 (1.19-1.87)	<.001
Education^a^		
Above median	1 [Reference]	[Reference]
Below median	1.06 (1.00-1.12)	.046
CDS		
0	1 [Reference]	[Reference]
1	1.58 (1.49-1.68)	<.001
≥2	2.69 (2.47-2.94)	<.001
Year		
For every 1 y increase	1.01 (1.00-1.03)	.07
Histology		
Ductal or lobular carcinoma	1 [Reference]	[Reference]
Other	0.98 (0.91-1.05)	.53
PR		
Positive	1 [Reference]	[Reference]
Negative	1.1 (1.02-1.18)	.01
RS		
0-25	1 [Reference]	[Reference]
>25	1.91 (1.77-2.05)	<.001
T staging		
1	1 [Reference]	[Reference]
2	1.52 (1.45-1.61)	<.001
3	2.34 (1.99-2.75)	<.001
N staging		
0	1 [Reference]	[Reference]
1a	1.61 (1.51-1.71)	<.001
Grade		
1	1 [Reference]	[Reference]
2	1.13 (1.07-1.21)	<.001
3	1.49 (1.37-1.61)	<.001
Other	0.86 (0.36-2.07)	.74
LVSI		
No	1 [Reference]	[Reference]
Yes	1.14 (1.07-1.23)	<.001
Chemotherapy		
No	1 [Reference]	[Reference]
Yes	0.71 (0.66-0.76)	<.001
Radiation		
No	1 [Reference]	[Reference]
Yes	0.59 (0.55-0.64)	<.001
Surgery		
Lumpectomy	1 [Reference]	[Reference]
Mastectomy	0.71 (0.65-0.77)	<.001
Other	1.46 (0.55-3.90)	.45
Margin		
Negative	1 [Reference]	[Reference]
Positive	1.19 (1.05-1.36)	.007

Abbreviations: aHR, adjusted hazards ratio; CDS, Charlson-Deyo comorbidity score; LVSI, lymphovascular space invasion; PR, progesterone receptor; RS, 21-gene recurrence score.

^a^ Median education level was 10.9% of adults living in a zip code who did not graduate from high school.

**Figure 1.**
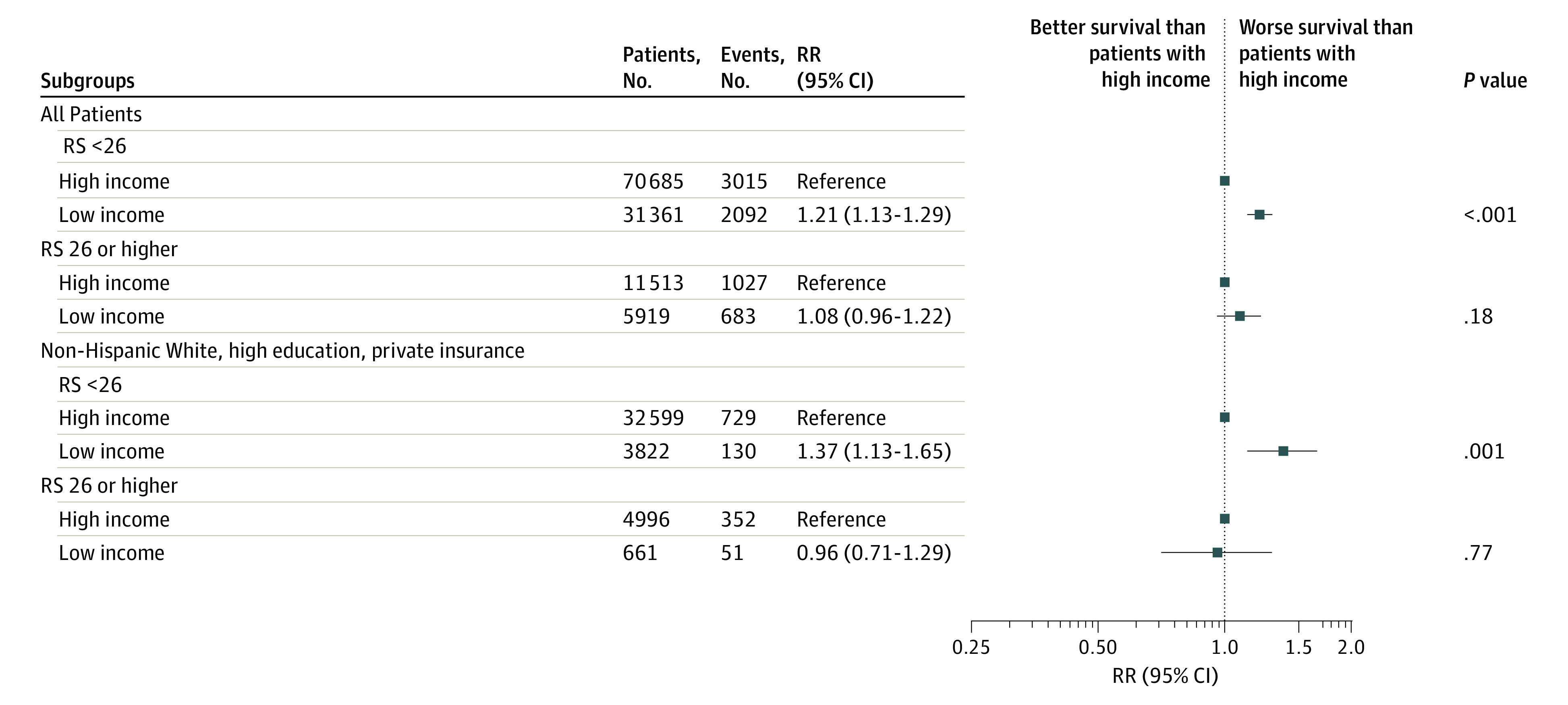
Forest Plot of Cox Multivariable Analysis for the Association of Survival and Income Levels Stratified by 21-Gene Recurrence Score RR indicates risk ratio; RS, 21-gene recurrence score.

**Figure 2.**
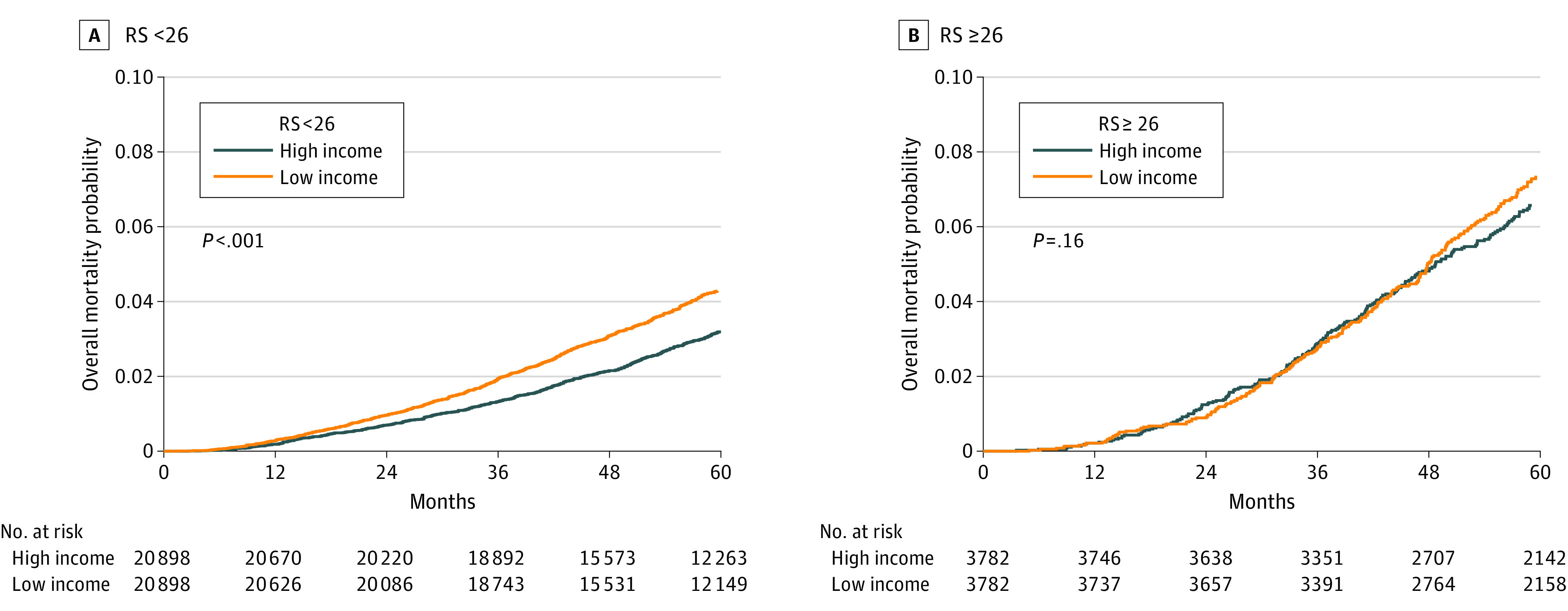
Cumulative Hazard of Overall Mortality Between High vs Low Income Stratified by 21-Gene Recurrence Score RS indicates 21-gene recurrence score.

On subgroup analysis, similar findings were noted among those with RS below 26, while there were no significant OS differences between income levels among others with RS 26 or higher (Figure 1). This finding was again observed among 20 898 and 3782 matched pairs for RS below 26 (HR, 1.36; 95% CI, 1.25-1.48; *P* < .001) and 26 or higher (HR, 1.11; 95% CI, 0.96-1.28; *P* = .16) (Figure 2; eTable 2 in Supplement 1).

On sensitivity analysis among non-Hispanic White women with high education and private medical insurance, logistic MVA similarly showed that, compared with high income, low income was associated with higher RS (aOR, 1.16; 95% CI, 1.05-1.28; *P* = .003). Cox MVA showed low income was associated with worse OS compared with high income (aHR, 1.24; 95% CI, 1.06-1.46; *P* = .007). The interaction term was statistically significant (interaction *P* = .03), with similar findings on subgroup analysis (Figure 1).

## Discussion

To our knowledge, this study using a nationwide oncology database has been the largest to suggest that low household income is associated with higher RS and worse survival outcome among those with RS below 26. Breast cancer stage and therapy are highly correlated with education level, race, and health insurance in the US.^2^ This prompted our sensitivity analysis that only included non-Hispanic White women with high education and private medical insurance. Our findings remained consistent in this subgroup.

Our main finding on the association between low income and more aggressive tumor biology is consistent with other population-based studies suggesting a higher incidence of triple-negative breast cancer among patients with low SES.^6,7,8^ Similar findings were also noted among Hispanic women as well.^16^ However, these studies utilized census tract–level SES and the Yost index, instead of education and income levels separately. In our study, income levels, but not education, were associated with RS. A lack of association between education levels and breast cancer risk in our study was consistent with several population-based^17^ and prospective cohort^18^ studies.

Rationales for the association between income levels and intrinsic tumor properties as indicated by RS in our study remain unclear. Low income levels may lead to less cancer screening with consequent delay in diagnosis and worsening of tumor biology.^3^ Moreover, financial distress has been shown to result in increased psychological and emotional distress, poor quality of life, and depression.^19^ Such distress may lead to dysregulation in stress pathways,^1,20^ reduced tumor suppressor p53 function,^21^ and more aggressive tumor biology with distant metastasis.^22^ High RS also includes genes responsible for tumor proliferation and invasion.^23^ The limited efficacy of chemotherapy with high RS^11,24^ may explain the lack of significant association between income levels and survival outcomes among patients with RS of 26 or above in our study. Further studies are warranted to investigate the pathways connecting financial distress and pathways for tumorigenesis.

### Limitations

This study had several limitations. Because of the retrospective nature of our study, some clinically relevant variables, such as performance status, tumor recurrence, breast cancer-specific survival, systemic therapy agents, and adherence to screening and treatments, were unavailable for analysis, which may have resulted in residual confounding despite matching and sensitivity analysis. In particular, most patients with unknown RS and/or income levels also had unknown education levels (eTable 1 in Supplement 1). Although education levels were not associated with high RS in our study (Table 2), the exclusion of such patients may have led to additional selection bias. Given the lack of patient-level income data, our findings may not be generalizable to individual patients from different socioeconomic backgrounds within the same zip code. In addition, given a small proportion of patients with high RS as shown in population-based studies,^25,26^ subgroup analysis in our study may be inadequately powered to detect OS differences among patients with high RS.

## Conclusions

In our observational cohort study, low household income was independently associated with higher RS and worse survival outcomes among those with RS below 26, but not RS 26 or higher. Further studies are warranted to investigate the mechanism behind the association between socioeconomic determinants of health and intrinsic tumor biology among patients with breast cancer.

## Notes

Supplement 1. **eTable 1.** Baseline Patient, Tumor, and Treatment Characteristics Between Those With Unknown Versus Known 21-gene Recurrence Score and/or Income Levels **eTable 2.** Baseline Patient, Tumor, and Treatment Characteristics After Matching

Supplement 2.
Data Sharing Statement

## References

[1] Williams DR, Mohammed SA, Shields AE. Understanding and effectively addressing breast cancer in African American women: Unpacking the social context. Cancer. 2016;122(14):2138-2149. doi:10.1002/cncr.29935 26930024 PMC5588632

[2] Bradley CJ, Given CW, Roberts C. Race, socioeconomic status, and breast cancer treatment and survival. J Natl Cancer Inst. 2002;94(7):490-496. doi:10.1093/jnci/94.7.490 11929949

[3] Silber JH, Rosenbaum PR, Ross RN, . Disparities in breast cancer survival by socioeconomic status despite Medicare and Medicaid insurance. Milbank Q. 2018;96(4):706-754. doi:10.1111/1468-0009.12355 30537364 PMC6287075

[4] Kalinsky K, Barlow WE, Gralow JR, . 21-gene assay to inform chemotherapy benefit in node-positive breast cancer. N Engl J Med. 2021;385(25):2336-2347. doi:10.1056/NEJMoa2108873 34914339 PMC9096864

[5] Sparano JA, Gray RJ, Makower DF, . Adjuvant chemotherapy guided by a 21-gene expression assay in breast cancer. N Engl J Med. 2018;379(2):111-121. doi:10.1056/NEJMoa1804710 29860917 PMC6172658

[6] Bauer KR, Brown M, Cress RD, Parise CA, Caggiano V. Descriptive analysis of estrogen receptor (ER)-negative, progesterone receptor (PR)-negative, and HER2-negative invasive breast cancer, the so-called triple-negative phenotype: a population-based study from the California cancer registry. Cancer. 2007;109(9):1721-1728. doi:10.1002/cncr.22618 17387718

[7] Qin B, Babel RA, Plascak JJ, . Neighborhood social environmental factors and breast cancer subtypes among Black women. Cancer Epidemiol Biomarkers Prev. 2021;30(2):344-350. doi:10.1158/1055-9965.EPI-20-1055 33234556 PMC7867587

[8] Rauscher GH, Campbell RT, Wiley EL, Hoskins K, Stolley MR, Warnecke RB. Mediation of racial and ethnic disparities in estrogen/progesterone receptor-negative breast cancer by socioeconomic position and reproductive factors. Am J Epidemiol. 2016;183(10):884-893. doi:10.1093/aje/kwv226 27076668 PMC4867153

[9] Bilimoria KY, Stewart AK, Winchester DP, Ko CY. The National Cancer Data Base: a powerful initiative to improve cancer care in the United States. Ann Surg Oncol. 2008;15(3):683-690. doi:10.1245/s10434-007-9747-3 18183467 PMC2234447

[10] Mallin K, Browner A, Palis B, . Incident cases captured in the National Cancer Database compared with those in U.S. population based central cancer registries in 2012-2014. Ann Surg Oncol. 2019;26(6):1604-1612. doi:10.1245/s10434-019-07213-1 30737668

[11] Ma SJ, Oladeru OT, Singh AK. Association of adjuvant chemotherapy with overall survival in patients with early-stage breast cancer and 21-gene recurrence scores of 26 or higher. JAMA Netw Open. 2020;3(5):e203876. doi:10.1001/jamanetworkopen.2020.3876 32364592 PMC7199111

[12] Ma SJ, Serra LM, Yu B, . Evaluation of risk stratification using gene expression assays in patients with breast cancer receiving neoadjuvant chemotherapy. Breast Cancer Res Treat. 2021;189(3):737-745. doi:10.1007/s10549-021-06269-6 34519904

[13] van ’t Veer LJ, Dai H, van de Vijver MJ, . Gene expression profiling predicts clinical outcome of breast cancer. Nature. 2002;415(6871):530-536. doi:10.1038/415530a 11823860

[14] Austin PC. Optimal caliper widths for propensity-score matching when estimating differences in means and differences in proportions in observational studies. Pharm Stat. 2011;10(2):150-161. doi:10.1002/pst.433 20925139 PMC3120982

[15] Haukoos JS, Lewis RJ. The Propensity Score. JAMA. 2015;314(15):1637-1638. doi:10.1001/jama.2015.13480 26501539 PMC4866501

[16] Banegas MP, Tao L, Altekruse S, . Heterogeneity of breast cancer subtypes and survival among Hispanic women with invasive breast cancer in California. Breast Cancer Res Treat. 2014;144(3):625-634. doi:10.1007/s10549-014-2882-1 24658879 PMC4045012

[17] Conroy SM, Shariff-Marco S, Koo J, . Racial/ethnic differences in the impact of neighborhood social and built environment on breast cancer risk: the Neighborhoods and Breast Cancer Study. Cancer Epidemiol Biomarkers Prev. 2017;26(4):541-552. doi:10.1158/1055-9965.EPI-16-0935 28196846 PMC5380527

[18] Palmer JR, Boggs DA, Wise LA, Adams-Campbell LL, Rosenberg L. Individual and neighborhood socioeconomic status in relation to breast cancer incidence in African-American women. Am J Epidemiol. 2012;176(12):1141-1146. doi:10.1093/aje/kws211 23171873 PMC3571232

[19] Tran G, Zafar SY. Financial toxicity and implications for cancer care in the era of molecular and immune therapies. Ann Transl Med. 2018;6(9):166. doi:10.21037/atm.2018.03.28 29911114 PMC5985271

[20] Antonova L, Aronson K, Mueller CR. Stress and breast cancer: from epidemiology to molecular biology. Breast Cancer Res. 2011;13(2):208. doi:10.1186/bcr2836 21575279 PMC3219182

[21] Feng Z, Liu L, Zhang C, . Chronic restraint stress attenuates p53 function and promotes tumorigenesis. Proc Natl Acad Sci U S A. 2012;109(18):7013-7018. doi:10.1073/pnas.1203930109 22509031 PMC3345015

[22] Chang A, Le CP, Walker AK, . β2-Adrenoceptors on tumor cells play a critical role in stress-enhanced metastasis in a mouse model of breast cancer. Brain Behav Immun. 2016;57:106-115. doi:10.1016/j.bbi.2016.06.011 27321906 PMC5060133

[23] Paik S, Shak S, Tang G, . A multigene assay to predict recurrence of tamoxifen-treated, node-negative breast cancer. N Engl J Med. 2004;351(27):2817-2826. doi:10.1056/NEJMoa041588 15591335

[24] Ma SJ, Oladeru OT, Farrugia M, . Survival outcome of adjuvant chemotherapy and high 21-gene recurrence score in early-stage breast cancer. Breast J. 2021;27(1):27-34. doi:10.1111/tbj.14130 33274486

[25] Cheng R, Kong X, Wang X, Fang Y, Wang J. Oncotype DX breast recurrence score distribution and chemotherapy benefit among women of different age groups with HR-positive, HER2-negative, node-negative breast cancer in the SEER database. Front Oncol. 2020;10:1583. doi:10.3389/fonc.2020.01583 33194568 PMC7663955

[26] Wang J, He ZY, Dong Y, Sun JY, Zhang WW, Wu SG. The distribution and outcomes of the 21-gene recurrence score in T1-T2N0 estrogen receptor-positive breast cancer with different histologic subtypes. Front Genet. 2018;9:638. doi:10.3389/fgene.2018.00638 30619463 PMC6304349

