# Supplementary material for: Association of Neighborhood-Level Household Income With 21-Gene Recurrence Score and Survival Among Patients With Estrogen Receptor–Positive Breast Cancer
Source: JAMA Netw Open. 2023 Feb 21;6(2):e230179. doi: 10.1001/jamanetworkopen.2023.0179 (PMC9945075; doi:10.1001/jamanetworkopen.2023.0179)
Supplement: Supplement 1. — eTable 1. Baseline Patient, Tumor, and Treatment Characteristics Between Those With Unknown Versus Known 21-gene Recurrence Score and/or Income Levels eTable 2. Baseline Patient, Tumor, and Treatment Characteristics After Matching [file jamanetwopen-e230179-s001.pdf]

Supplemental Online Content

Ma SJ, Gill J, Waldman O, et al. Association of neighborhood-level household income with 21-gene recurrence score and survival among patients with estrogen receptor–positive breast cancer. *JAMA Netw Open.* 2023;6(2):e230179. doi:10.1001/jamanetworkopen.2023.0179

eTable 1. Baseline Patient, Tumor, and Treatment Characteristics Between Those With Unknown Versus Known 21-gene Recurrence Score and/or Income Levels

eTable 2. Baseline Patient, Tumor, and Treatment Characteristics After Matching

This supplemental material has been provided by the authors to give readers additional information about their work.

© 2023 Ma SJ et al. *JAMA Network Open.*

eTable 1. Baseline patient, tumor, and treatment characteristics between those with unknown versus known 21-gene recurrence score and/or income levels

	Known RS and income		Unknown RS or income	
	N	%	N	%
Age				
<50 years	23255	19.5	6703	18.6
50 years or older	96223	80.5	29381	81.4
Facility				
Nonacademic	78414	65.6	25200	69.8
Academic	37882	31.7	9978	27.7
Not available	3182	2.7	906	2.5
Race				
Non-Hispanic White	98270	82.2	29868	82.8
Hispanic	7245	6.1	2042	5.7
Black	9226	7.7	2933	8.1
Asian/Pacific Islander	4737	4.0	1241	3.4
Insurance				
None	1360	1.1	460	1.3
Private	71281	59.7	21403	59.3
Government	45637	38.2	13811	38.3
Not available	1200	1.0	410	1.1
Education				
Above median	76195	63.8	7793	21.6
Below median	43283	36.2	5724	15.9
Not available	0	0.0	22567	62.5
CDS				
0	101331	84.8	30155	83.6
1	14610	12.2	4741	13.1
2+	3537	3.0	1188	3.3
Year				
2006-2013	48068	40.2	15860	44.0
2014-2017	70706	59.2	20034	55.5

© 2023 Ma SJ et al. *JAMA Network Open.*

Histology				
Ductal or lobular carcinoma	102769	86.0	30875	85.6
Other	16709	14.0	5209	14.4
PR				
Positive	108168	90.5	33163	91.9
Negative	11310	9.5	2921	8.1
T staging				
1	89114	74.6	27088	75.1
2	28669	24.0	8507	23.6
3	1695	1.4	489	1.4
N staging				
0	101467	84.9	31377	87.0
1a	18011	15.1	4707	13.0
Grade				
1	33284	27.9	10471	29.0
2	63708	53.3	19286	53.4
3	18112	15.2	4882	13.5
Other	70	0.1	19	0.1
Not available	4304	3.6	1426	4.0
LVSI				
No	91324	76.4	28385	78.7
Yes	14644	12.3	3840	10.6
Not available	13510	11.3	3859	10.7
Chemotherapy				
No	94261	78.9	29797	82.6
Yes	25217	21.1	6287	17.4
Radiation				
No	37178	31.1	10842	30.0
Yes	80970	67.8	24826	68.8
Not available	1307	1.1	393	1.1

© 2023 Ma SJ et al. *JAMA Network Open.*

Surgery				
Lumpectomy	80787	67.6	24782	68.7
Mastectomy	38661	32.4	11295	31.3
Other	31	0.0	7	0.0
Margin				
Negative	115414	96.6	34913	96.8
Positive	3631	3.0	1037	2.9
Not available	433	0.4	134	0.4

N: number, CDS: Charlson-Deyo comorbidity score; PR: progesterone receptor; RS: 21-gene recurrence score; LVSI: lymphovascular space invasion

© 2023 Ma SJ et al. *JAMA Network Open.*

eTable 2. Baseline patient, tumor, and treatment characteristics after matching

	RS < 26					RS 26 or higher				
	High income		Low income			High income		Low income		
	N	%	N	%	SMD	N	%	N	%	SMD
Age					0.002					0.014
<50 years	3789	18.1	3775	18.1		655	17.3	675	17.8	
50 years or older	17109	81.9	17123	81.9		3127	82.7	3107	82.2	
Facility					0.007					0.004
Nonacademic	14319	68.5	14252	68.2		2534	67.0	2523	66.7	
Academic	6079	29.1	6168	29.5		1106	29.2	1114	29.5	
Not available	500	2.4	478	2.3		142	3.8	145	3.8	
Race					0.02					0.005
Non-Hispanic White	17044	81.6	16886	80.8		2901	76.7	2910	76.9	
Hispanic	1593	7.6	1572	7.5		299	7.9	288	7.6	
Black	1583	7.6	1812	8.7		447	11.8	458	12.1	
Asian/Pacific Islander	678	3.2	628	3.0		135	3.6	126	3.3	
Insurance					0.02					0.041
None	263	1.3	295	1.4		50	1.3	58	1.5	
Private	11937	57.1	11625	55.6		2188	57.9	2088	55.2	
Government	8508	40.7	8770	42.0		1496	39.6	1588	42.0	
Not available	190	0.9	208	1.0		48	1.3	48	1.3	
Education					0.001					0.004
Above median	8051	38.5	8037	38.5		1467	38.8	1460	38.6	
Below median	12847	61.5	12861	61.5		2315	61.2	2322	61.4	
CDS					0.01					0.036
0	17453	83.5	17381	83.2		3187	84.3	3128	82.7	
1	2781	13.3	2822	13.5		471	12.5	524	13.9	
2+	664	3.2	695	3.3		124	3.3	130	3.4	
Year					0.009					0.02
2006-2013	8378	40.1	8393	40.2		1552	41.0	1571	41.5	
2014-2018	12417	59.4	12388	59.3		2193	58.0	2181	57.7	

© 2023 Ma SJ et al. *JAMA Network Open.*

Not available	103	0.5	117	0.6		37	1.0	30	0.8	
Histology					0.015					0.014
Ductal or lobular carcinoma	17989	86.1	17882	85.6		3432	90.7	3417	90.3	
Other	2909	13.9	3016	14.4		350	9.3	365	9.7	
PR					0.005					0.015
Positive	19679	94.2	19654	94.0		2681	70.9	2656	70.2	
Negative	1219	5.8	1244	6.0		1101	29.1	1126	29.8	
T staging					0.008					0.001
1	15787	75.5	15733	75.3		2427	64.2	2436	64.4	
2	4812	23.0	4843	23.2		1310	34.6	1294	34.2	
3	299	1.4	322	1.5		45	1.2	52	1.4	
N staging					0.009					0.023
0	17688	84.6	17618	84.3		3309	87.5	3280	86.7	
1a	3210	15.4	3280	15.7		473	12.5	502	13.3	
Grade					0.008					0.015
1	6431	30.8	6534	31.3		316	8.4	307	8.1	
2	11707	56.0	11533	55.2		1570	41.5	1572	41.6	
3	2050	9.8	2078	9.9		1780	47.1	1777	47.0	
Other	11	0.1	10	0.0		7	0.2	8	0.2	
Not available	699	3.3	743	3.6		109	2.9	118	3.1	
RS					0.01					<0.001
0-15	11970	57.3	12073	57.8		0	0.0	0	0.0	
16-25	8928	42.7	8825	42.2		0	0.0	0	0.0	
>25	0	0.0	0	0.0		3782	100.0	3782	100.0	
LVSI					0.002					0.013
No	16091	77.0	16012	76.6		2667	70.5	2654	70.2	
Yes	2258	10.8	2322	11.1		609	16.1	639	16.9	
Not available	2549	12.2	2564	12.3		506	13.4	489	12.9	
Chemotherapy					0.008					0.004
No	18492	88.5	18441	88.2		1003	26.5	1009	26.7	
Yes	2406	11.5	2457	11.8		2779	73.5	2773	73.3	

© 2023 Ma SJ et al. *JAMA Network Open.*

Radiation					0.004					0.003
No	6424	30.7	6586	31.5		1161	30.7	1212	32.0	
Yes	14248	68.2	14092	67.4		2572	68.0	2519	66.6	
Not available	226	1.1	220	1.1		49	1.3	51	1.3	
Surgery					0.018					0.024
Lumpectomy	14264	68.3	14087	67.4		2571	68.0	2530	66.9	
Mastectomy	6630	31.7	6807	32.6		1210	32.0	1250	33.1	
Other	4	0.0	4	0.0		1	0.0	2	0.1	
Margin					0.005					0.013
Negative	20230	96.8	20200	96.7		3662	96.8	3649	96.5	
Positive	601	2.9	625	3.0		107	2.8	117	3.1	
Not available	67	0.3	73	0.3		13	0.3	16	0.4	

N: number, SMD: standard mean difference; CDS: Charlson-Deyo comorbidity score; PR: progesterone receptor; RS: 21-gene recurrence score; LVSI: lymphovascular space invasion

© 2023 Ma SJ et al. *JAMA Network Open.*
