# Supplementary material for: Association of Neighborhood-Level Household Income With 21-Gene Recurrence Score and Survival Among Patients With Estrogen Receptor–Positive Breast Cancer
Source: JAMA Netw Open. 2023 Feb 21;6(2):e230179. doi: 10.1001/jamanetworkopen.2023.0179 (PMC9945075; doi:10.1001/jamanetworkopen.2023.0179)
Supplement: Supplement 2. — Data Sharing Statement [file jamanetwopen-e230179-s002.pdf]

# Data Sharing Statement

Ma. Association of Neighborhood-Level Household Income With 21-Gene Recurrence Score and Survival Among Patients With Estrogen Receptor-Positive Breast Cancer. *JAMA Netw Open*. Published February 21, 2023. doi:10.1001/jamanetworkopen.2023.0179

**Data**
**Data available:** No

**Additional Information**
**Explanation for why data not available:** Database is publicly available through the American College of Surgeons.
